# Trawl impacts on the relative status of biotic communities of seabed sedimentary habitats in 24 regions worldwide

**DOI:** 10.1073/pnas.2109449119

**Published:** 2022-01-04

**Authors:** C. Roland Pitcher, Jan G. Hiddink, Simon Jennings, Jeremy Collie, Ana M. Parma, Ricardo Amoroso, Tessa Mazor, Marija Sciberras, Robert A. McConnaughey, Adriaan D. Rijnsdorp, Michel J. Kaiser, Petri Suuronen, Ray Hilborn

**Affiliations:** ^a^Oceans and Atmosphere, Commonwealth Scientific and Industrial Research Organisation, Brisbane, QLD 4067, Australia;; ^b^School of Ocean Sciences, Bangor University, Menai Bridge LL59 5AB, United Kingdom;; ^c^Lowestoft laboratory, Centre for Environment, Fisheries, and Aquaculture Science, Lowestoft NR33 0HT, United Kingdom;; ^d^Graduate School of Oceanography, University of Rhode Island, Narragansett, RI 02882;; ^e^Centro Nacional Patagónico, Consejo Nacional de Investigaciones Científicas y Técnicas, Puerto Madryn 9120, Argentina;; ^f^School of Aquatic and Fishery Sciences, University of Washington, Seattle, WA 98195;; ^g^Biodiversity, Environment and Climate Change, Department of Environment Land Water and Planning, East Melbourne, VIC 3002, Australia;; ^h^The Lyell Centre, Heriot-Watt University, Edinburgh EH14 4AS, United Kingdom;; ^i^Alaska Fisheries Science Center, National Oceanic and Atmospheric Administration, Seattle, WA 98115;; ^j^Wageningen Marine Research, Wageningen University and Research, IJmuiden 1976 CP, Netherlands;; ^k^Fisheries and Aquaculture Department, Food and Agriculture Organization of the United Nations, Rome 00153, Italy;; ^l^Fisheries and fish resources, Natural Resources Institute Finland (Luke), Helsinki 00790, Finland

**Keywords:** trawl impacts, trawl footprints, recovery, habitat sensitivity, spatial upscaling

## Abstract

Bottom trawling is widespread globally and impacts seabed habitats. However, risks from trawling remain unquantified at large scales in most regions. We address these issues by synthesizing evidence on the impacts of different trawl-gear types, seabed recovery rates, and spatial distributions of trawling intensity in a quantitative indicator of biotic status (relative amount of pretrawling biota) for sedimentary habitats, where most bottom-trawling occurs, in 24 regions worldwide. Regional average status relative to an untrawled state (=1) was high (>0.9) in 15 regions, but <0.7 in three (European) regions and only 0.25 in the Adriatic Sea. Across all regions, 66% of seabed area was not trawled (status = 1), 1.5% was depleted (status = 0), and 93% had status > 0.8. These assessments are first order, based on parameters estimated with uncertainty from meta-analyses; we recommend regional analyses to refine parameters for local specificity. Nevertheless, our results are sufficiently robust to highlight regions needing more effective management to reduce exploitation and improve stock sustainability and seabed environmental status—while also showing seabed status was high (>0.95) in regions where catches of trawled fish stocks meet accepted benchmarks for sustainable exploitation, demonstrating that environmental benefits accrue from effective fisheries management. Furthermore, regional seabed status was related to the proportional area swept by trawling, enabling preliminary predictions of regional status when only the total amount of trawling is known. This research advances seascape-scale understanding of trawl impacts in regions around the world, enables quantitative assessment of sustainability risks, and facilitates implementation of an ecosystem approach to trawl fisheries management globally.

## Significance

We estimated the biological state of seabed sedimentary habitats, with specified uncertainty, in 24 trawled regions worldwide. Seabed status differed greatly among regions (from 0.25 to 0.999, relative to an untrawled state of 1); 15 regions had average status > 0.9. Two-thirds of all assessed seabed area was untrawled with status = 1, 93% had status > 0.8, but 1.5% had status = 0. The total area swept by trawling was a strong driver of regional status, providing a relationship to predict status from the regional estimated total amount of trawling. Seabed status is high in regions where fisheries are exploited sustainably—emphasizing that good fishery management contributes to better ecosystem outcomes—and, conversely, low status highlights regions needing improved management.

Bottom-trawl fishing occurs worldwide and is the most extensive anthropogenic direct physical disturbance to seabed habitats (1, 2). Towing trawl gear such as otter or beam trawls or dredges along the seabed has a wide range of direct and indirect impacts on habitats, the broader ecosystem, and the services they provide (3–8) and often is portrayed as a destructive fishing practice by some environmental nongovernmental organizations. However, bottom-trawl fisheries provide about a quarter of marine catch (9), making substantial contributions to global food supply and livelihoods (10). Recognition of the wider environmental consequences of fishing, including seabed impacts of trawling, has contributed to the development of an “ecosystem approach to fisheries” [EAF (11)] that considers broader ecosystem sustainability in balance with fishery production when managing fisheries. EAF principles are being adopted widely into international and national policy commitments, fishery management plans, and sustainable-seafood certifications (12).

Balancing fishery production and ecosystem sustainability, however, remains a globally challenging issue—partly because the required indicators of ecosystem state often are unavailable or cost prohibitive to acquire at management scales. Consequently, a common approach has been to consider the risks of fishing impacts using expert judgement and/or qualitative scoring approaches, which provide indicators of relative risk (13–15). In contrast, quantitative methods provide continuous objective indicators of ecosystem state, more useful for supporting management of fishery impacts under EAF (13–17). Quantitative methods require appropriate response indicators. In an evaluation of seven candidate indicators (18), total seabed community abundance (biomass and numbers of individuals) was the best-performing indicator of seabed state, meeting all nine criteria required for state indicators (19) and also relating directly to ecosystem functioning (15, 18).

The implementation of EAF for bottom-trawl fisheries requires assessment of their impacts on the status of communities of seabed biota. We address this global challenge for EAF by quantifying a community abundance state indicator for seabed sedimentary (benthic) habitats on continental shelves and slopes in 24 large regions covering 7.92 million km^2^ worldwide, accounting for 18.9% of the 0- to 1,000-m depth range (20) and 19.5% of all trawl landings (9) globally. We synthesize the required information regarding the direct impacts of trawling and recovery rates (7, 8), distribution and intensity of bottom trawling (9), and mapped composition of seabed sediments (e.g., ref. 21) in a quantitative model of the relative benthic status (RBS) of the seabed (14), recently recommended as the best performing of three quantitative indicators evaluated (15). We focused on sedimentary habitats because they comprise the majority area of seabed [>99% of shelf and slope has a >1-m layer of sediments (22)], most bottom trawling occurs on these habitats, and managers require these fisheries to be assessed and to be sustainable.

The RBS model estimates the eventual abundance of biota relative to their untrawled abundance as a function (Eq. 1) of trawl depletion rates (proportional reduction per trawl pass), recovery rates (maximum annual increase in proportional abundance), and current chronic trawling-intensity levels (as swept-area ratio, SAR). We quantified RBS and its uncertainty in high-resolution grid cells (∼1 km^2^) in each region—after first updating the previous series of meta-analyses used to estimate depletion and recovery rates (7) with additional data (see *Methods*). We also estimate regional mean RBS as the average of grid cell values to provide a relative indicator of the overall state of regional seabeds.

## Results

### Parameter Estimates.

We estimated trawl depletion rates of benthic communities in mud, sand, and gravel habitats for each trawl gear type by remodeling the relationship (7) between depletion rates of biota (*SI Appendix*, Table S1) and seabed penetration depths (PD) of different trawl gears, including some additional data and adding different sediment habitat types as a factor (*SI Appendix*, Table S2 and Fig. S1). Average depletion rates ranged from 0.047 to 0.261 depending on gear and habitat (*SI Appendix*, Table S3 and Fig. S2). Otter trawls caused the lowest depletion followed by beam trawls and towed dredges. Depletion rates were lower in sand than in gravel and mud.

We estimated recovery rates of benthic communities in sedimentary habitats by reanalyzing the relationship (7) of decreasing community relative abundance (as a combination of biomass and numbers of epifauna and infauna) along a gradient of increasing trawling impact (Eq. 2) with some additional data and including how the relationship depended on sediment types (*SI Appendix*, Fig. S3*A* and Table S4). Recovery rates were estimated (using the fitted model, Eq. 3) for an untrawled community so that RBS would indicate the state of community compositions that existed on and in sediments prior to trawling, including some slower-growing, larger-bodied, and longer-lived biota that are more sensitive to trawling. Average recovery rates ranged from 0.29 to 0.68 (lower confidence limits, CLs = 0.25 to 0.48) along a gravel to mud gradient (*SI Appendix*, Fig. S3*B*). Slower recovery with increasing gravel reflects the greater proportions of longer-lived species found in more stable gravel habitats (23, 24). We used the mean and lower CL of recovery estimates, representing a spectrum of more sensitive biota compositions, because of the higher level of concern for sensitive biota. These rates correspond to a range of maximum longevities (see figure 3 in ref. 25) averaging 8 y in mud to 18 y in gravel (and up to 11 to 22 y, respectively, for lower CL recovery rates). Longevities in sand were intermediate (mean = 10 y, up to 14 y for lower CL recovery).

Trawl SAR intensities of otter trawling, beam trawling, and towed dredging, mapped for grid cells covering 24 regions for which adequate trawling data were available (9), differed greatly among cells (0 to 210 y^−1^, mean = 0.42) as well as among regions (regional average SAR: range = 0.005 to 11 y^−1^, mean = 1.28, *SI Appendix*, Table S5). SAR was aggregated among cells at larger scales, but, at fine scales within small grid cells, most trawling tends to be distributed approximately randomly (26, 27), producing a dynamic mosaic of recently impacted, recovering, and undisturbed patches of seabed. Long-term, however, all patches are expected to be trawled at the average SAR of each grid cell (27, 28).

We assigned trawl depletion and recovery rates appropriate to the trawl gear and sediment type (*SI Appendix*, Fig. S4) of each grid cell, mapped for each region using available sediment data (*SI Appendix*, Table S5). We used these rates with the grid cell trawl SAR intensity values for each gear type in Eq. 1 to estimate cumulative trawl impacts and RBS for each grid cell and region (*SI Appendix*, Table S5) under current distributions of fishing. Grid cell RBS values range between 0 to 1; trawled cells have RBS < 1, and untrawled cells have RBS = 1. The mean RBS estimate represents a linear relative index of benthic state for sedimentary habitats, corresponding to effects on biota that have an average sensitivity to trawling (among the range of sensitivities comprising typical communities in these habitats prior to trawling). The lower CL of RBS is indicative of status for biota types having upper CL sensitivity. Thus, mean RBS = 0 does not imply that all biota are depleted; rather, that among the mix of biota present before trawling, those with average or greater sensitivity to trawling (the response indicated by RBS herein) would be entirely depleted, whereas more resilient types may remain.

### Regional Status.

**Fig. 1.**
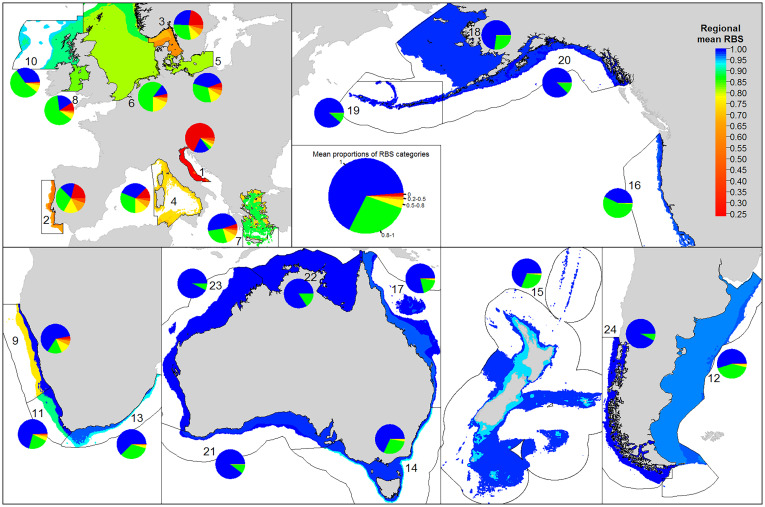
Maps of regional average RBS for continental shelves (0- to 200-m depth) and slopes (200- to 1,000-m) in 24 regions. Pie charts show proportional area by region in six RBS category intervals; the pie legend (*Center*) also indicates the average of category proportions across all regions. Black boundaries indicate study regions (i.e., exclusive economic zones or fishery management jurisdictions or large marine ecosystems). Region numbers and names follow Fig. 2.

Regional RBS was lower in most European regions and higher in most non-European regions (range 0.247 to 0.999; Fig. 1 maps). Average RBS was <0.7 in three European regions: the Adriatic Sea (0.25), west of Iberia (0.60), and Skagerrak–Kattegat (0.63). These three regions also had the highest percentage of area where RBS = 0 (68, 21, and 23% respectively; Fig. 1 pie charts; *SI Appendix*, Table S5). The European regions had <50% untrawled area where RBS = 1; lowest were the North Sea (11%), west of Iberia (16%), the Adriatic Sea (17%), and the Irish Sea (18%). All non-European regions except Northern Benguela had an average RBS ≥ 0.95. Chile, Australasia, and Alaska had the highest average RBS and the largest untrawled areas (68 to 93%).

In 17 regions, particularly in Europe, the RBS of continental shelves was lower than that of slopes (Fig. 1). Conversely, continental slopes had a lower mean RBS than shelves for seven regions outside of Europe, particularly southern Africa and southeast Australia. These differences primarily reflect distributions of trawling in shelf or slope areas (9).

**Fig. 2.**
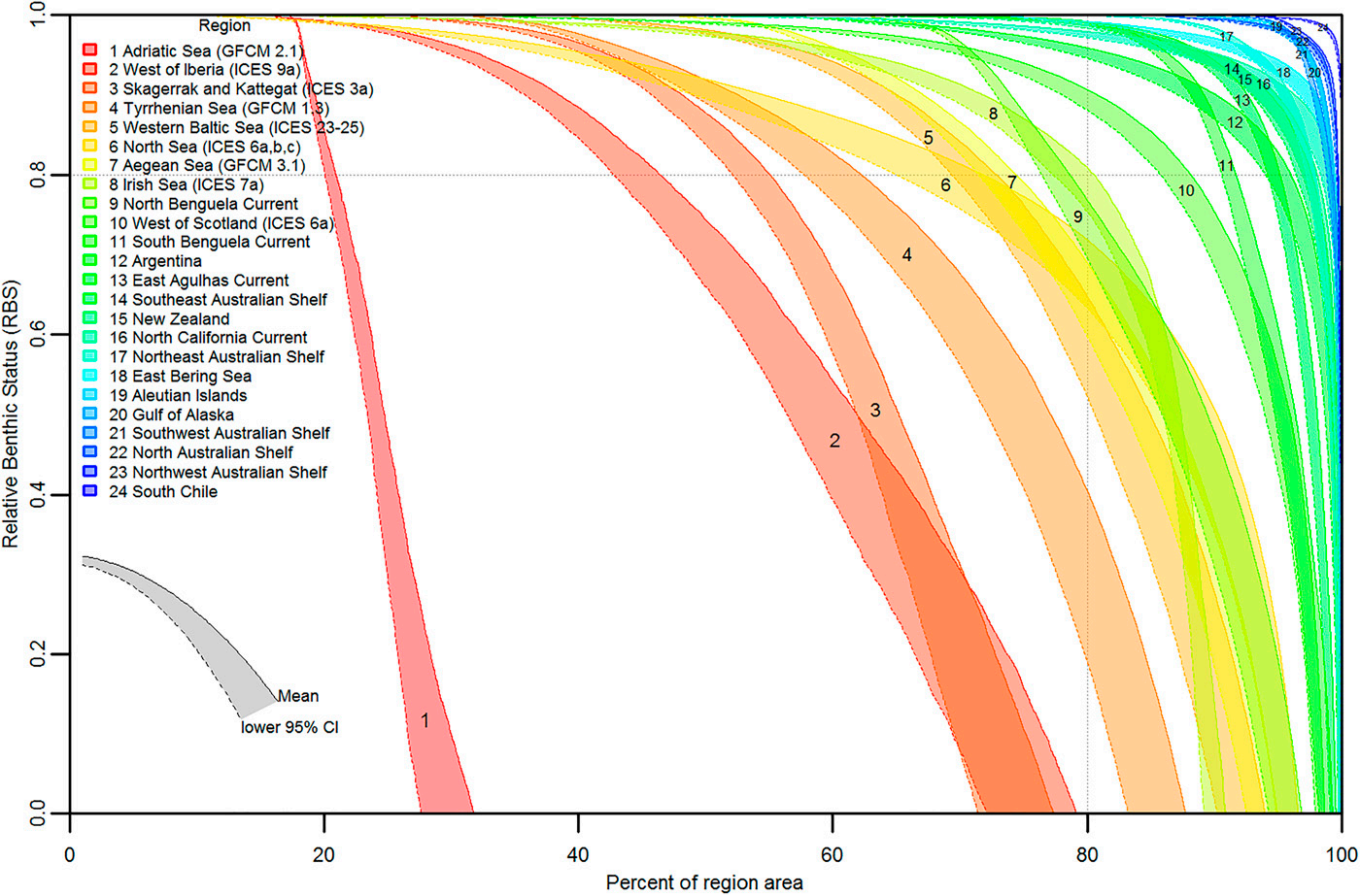
Distributions of grid cell RBS values (ordered 1 through 0) versus cumulative percentage of regional area. Where RBS = 1 at top/left indicates untrawled seabed, and RBS = 0 at bottom/right indicates depleted seabed. The lower uncertainty interval is indicated by the band between cell mean RBS and the lower 95% CL of cell RBS. Dotted horizontal and vertical lines at RBS = 0.8 and 80% of region area indicate example thresholds. The regions legend is ordered by regional average RBS.

Relationships between regional grid cell RBS values (in decreasing order) and cumulative seabed area (Fig. 2) show the proportion of seabed having any given status, provide more nuanced information about grid cell RBS than the regional average, and reflect spatial patterns of trawl impacts on different habitats. The regional area at the point of departure from RBS = 1 indicates the relative size of untrawled versus trawled seabed, and the areas under the curves correspond to average RBS. The percentage of regional areas where RBS = 0 indicates a seabed depleted of pretrawling biota that have average or higher sensitivity to trawling. Steeper curves reflect areas where trawling is more concentrated. For example, northern and southern Benguela have relatively short, steep upper curves compared to other regions with similar average RBS because fisheries in these regions target a narrow depth band on the slope (*cf*. Fig. 1). Longer, flatter upper curves reflect widespread low-intensity trawling. For example, the North Sea has the smallest percentage of untrawled area but also the smallest percentage of depleted seabed among European regions except west of Scotland. The Adriatic stands out with the lowest status and a very steep and almost linear RBS curve, indicating that most trawlable ground in the Adriatic is heavily trawled.

The uncertainty intervals of RBS curves (Fig. 2) arise from the use of the mean and lower CL of estimated recovery rates, representing a spectrum of more sensitive sedimentary biota; hence, they also indicate potential outcomes for a range of biota with recovery rates corresponding to maximum longevities of about 8 to 22 y. The majority of biota comprising seabed communities in sedimentary habitats are shorter lived and more resilient (25); thus, the RBS uncertainty interval presented in Fig. 2 represents a more precautionary range of RBS outcomes.

RBS with uncertainty intervals can be used to frame risk assessments for trawling impacts. For example, if, as part of regional environmental objectives, an appropriate threshold for acceptable seabed status is defined, then RBS curves with uncertainty intervals can estimate the probability of status being above or below that threshold, thus informing the risk of environmental objectives not being achieved. As an illustration, if an acceptable threshold is set at RBS > 0.8 for >80% of a regional area (Fig. 2), then, in the case of the North Sea (region 6), the lower and upper 95% CLs for RBS at 80% of regional area are 0.633 and 0.790 (mean = 0.719), and the lower and upper CLs for the percentage of regional area having RBS = 0.8 are 65.0 and 78.9% (mean = 71.8%). Thus, the 95% CI for RBS is just below the illustrative 0.8-at-80%-area threshold and, therefore, there is >97.5% probability that seabed status would not meet a threshold set at that level. Similarly, six other regions (1–5, 7) have >97.5% probability of not meeting this threshold; northern Benguela has more than ∼50% probability and the Irish Sea almost 50% probability. Conversely, of the 24 regions, 15 would have <2.5% probability of not meeting the example objective (i.e., the lower CL for their RBS curves are above the 0.8-at-80%-area threshold).

**Fig. 3.**
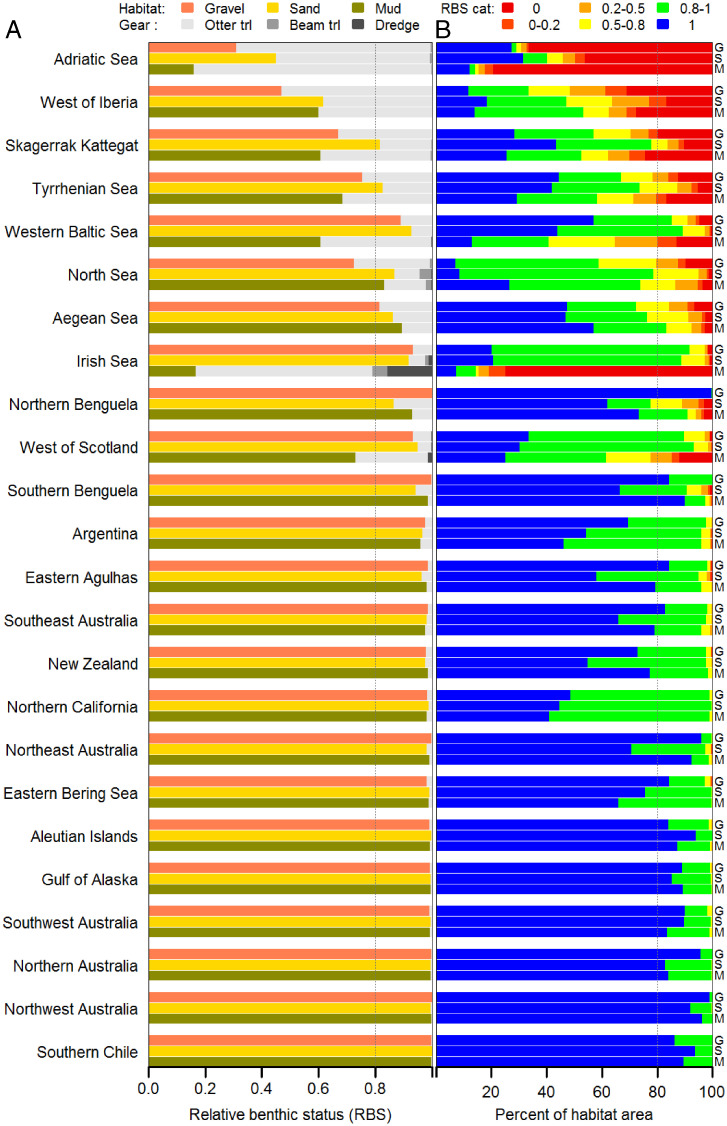
Bar plots of (*A*) average RBS for gravel, sand, and mud (G, S, and M; colored bars) habitats within regions, and reduction of RBS (=1 − RBS) because of cumulative impacts of different trawl gear types (stacked gray bars); (*B*) percentage area of each regional habitat in six RBS category intervals. Vertical dotted line indicates RBS = 0.8 in *A* and 80% of regional habitat area in *B*.

The differing status of gravel, sand, and mud habitats (Fig. 3) reflects both their differing sensitivity to trawling (*SI Appendix*, Fig. S4) and the distribution of trawling. Within regions, sand habitats typically have higher average RBS and smaller proportions of low RBS categories because of their lower sensitivity than mud or gravel. The biggest within-region differences among habitats are in the Irish Sea, where mud status is heavily reduced. Overall, eight regions have one or more habitats with average RBS < 0.8 (14 habitats in total, Fig. 3*A*); 21 habitats in 10 regions have <80% of area with RBS > 0.8, whereas 51 habitats in 14 regions are above this threshold (Fig. 3*B*). Otter trawling is the most widespread and greatest contributor to cumulative reductions of RBS (Fig. 3*A*), even though other gear types cause greater depletion per trawl pass. Beam trawling noticeably reduces RBS in the North Sea, as does dredging in the Irish Sea.

**Fig. 4.**
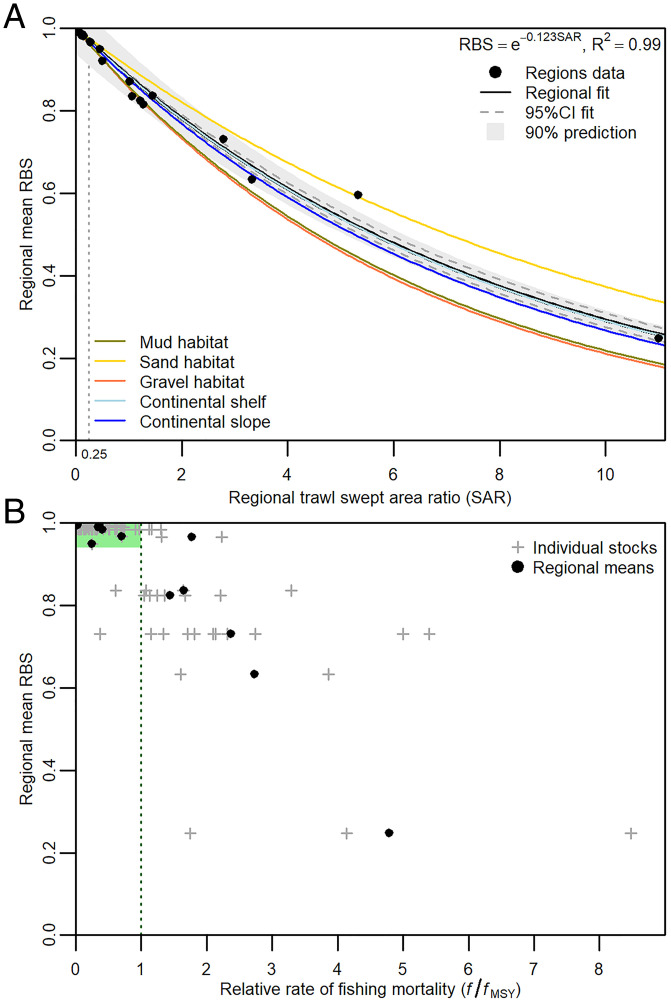
Relationships for regional average RBS versus (*A*) regional SAR for all 24 regions, fitted relationship and prediction interval, with fitted relationships for sedimentary habitats and continental shelves and slopes; vertical dotted line indicates SAR = 0.25 (see text); and (*B*) stock exploitation as the ratio of *f* over *f*_MSY_ reference point for individual trawl fishery stocks in 12 regions for years 2010, 2011, and 2012 (9) and regional average *f*/*f*_MSY_; green vertical dotted line at *f*/*f*_MSY_ = 1 indicates an accepted sustainable upper limit on fishing rate; light-green shading emphasizes data for regions where most stocks are managed sustainably (*f*/*f*_MSY_ < 1) and average RBS ≥ 0.95; linear fit to all 87 stocks in 12 regions: slope = −0.101, R^2^ = 0.71, *P* < 0.001; linear fit to 12 regional means: slope = −0.131, R^2^= 0.91, *P* < 0.001.

The total amount of trawling in a region is a strong driver of regional RBS (Fig. 4*A*). For regions that lack high-resolution spatial data for trawling and habitats, this relationship can be used to predict regional RBS with specifiable uncertainty from information about the total amount of trawling and gear types used. Such predictions underestimate RBS for sand but overestimate RBS for gravel and mud habitats (Fig. 4*A*). They may also underestimate RBS for tropical regions, where recovery rates might be faster, but, hence, would be conservative. This approach can infer preliminary regional status and facilitate prioritization of management needs, including in regions with higher levels of trawling effort such as Southeast Asia. Furthermore, Amoroso et al. (9) showed that where fishing exploitation is at or below that needed to catch maximum sustainable yield (MSY: a widely accepted reference point for sustainable fisheries), regional SAR was ≤0.25. Here, where regional SAR is ≤0.25, the average RBS is 95% likely to be >0.91 (Fig. 4*A*). The average RBS is >0.91 for 15 of 24 regions. We also directly compare average regional RBS and an accepted indicator of the exploitation status of fish stocks (the ratio of fishing mortality *f* relative to the maximum sustainable fishing mortality *f*_MSY_, see *Methods*) (Fig. 4*B*). There was a clear, though scattered, negative relationship between regional RBS and the ratio *f/f*_MSY_ of stocks. In regions where most stocks are managed sustainably (i.e., *f/f*_MSY_ < 1), the average regional RBS is >0.95, suggesting that managing trawl fisheries for sustainable exploitation of fish stocks contributes substantially toward ensuring that seabed status is high.

While assessing the status of sedimentary habitats is critical to ensuring integrity of the majority area of seabed ecosystems at the broadest scales, perhaps more concern surrounds rarer, more sensitive biogenic habitat types. However, suitable high-resolution data on the distribution and sensitivity for such habitats were not available for all our 24 regions. Hence, although we took a precautionary approach by using recovery rates applicable to pretrawling seabed community compositions and considering the lower CI of RBS, we could not assess highly sensitive habitat-forming biota types that can characterize vulnerable marine ecosystems [VMEs (29, 30)]. VME biota typically have distributions restricted to hard grounds, which may have low exposure to trawling (31, 32), but they also have high trawl depletion and slow recovery rates—hence management seeks to prevent impacts on VMEs (29). RBS can be applied to VME biota; however, the scarcity of data for their distribution, depletion, and recovery must be addressed first—consequently, there are few cases in which their regional status has been assessed (e.g., refs. 31 and 33 using a dynamic Schaefer model and ref. 34 using RBS). Here, in lieu of assessing RBS for habitat-forming biota, we calculated the percentage of each region where trawl SAR exceeded an estimated local extinction threshold for highly sensitive biota (at SAR > 0.35, *SI Appendix*, Fig. S5*A*). This ranges from 0.2% of seabed area in southern Chile to 82% in the Adriatic Sea and is >20% for 10 regions (all European regions and northern Benguela). Areas for this metric are very similar to areas of regional “uniform” trawl footprints as estimated by Amoroso et al. (9) (*SI Appendix*, Fig. S5 *A* and *B*). Where they do differ (*SI Appendix*, Fig. S5*B*), the threshold SAR needed to categorize cells as trawled that yields an equivalent area as the uniform footprint remains indicative of extinction thresholds for highly sensitive biota (*SI Appendix*, Fig. S5*C*). Thus, the area of the uniform footprint also corresponds closely to the area of regions where highly sensitive biota cannot persist. Furthermore, we also calculated that the percentage of each region where trawl SAR was <0.07, allowing highly sensitive biota to maintain a status >0.8, ranges from 18% of seabed area in the Adriatic to 98% in southern Chile (*SI Appendix*, Fig. S5*A*) and is >80% for 10 non-European regions.

## Discussion and Conclusions

We used a quantitative indicator of RBS to synthesize recent advances in the understanding of trawling disturbance to the seabed and provide a seascape-scale assessment of cumulative trawl impacts on the relative biotic state of seabed sedimentary habitats, where most trawling occurs, under current levels of chronic fishing. Our results give insight into the sustainability of bottom trawling in 24 diverse regions around the world and provide comparisons for guiding regional management of environmental risks from trawling.

The RBS method is based on an established population dynamics model, widely applied in ecology and for fisheries assessments, and has been recommended as the best performing method to assess trawling impacts in sedimentary habitats (15). In our application to sedimentary habitats, RBS estimated status based on relative abundance of combined seabed community biomass and numbers, which have been shown to be the most suitable indicators of bottom-trawling impacts (18), as they respond strongly to trawling, perform well against nine criteria for indicators (19), relate directly to ecosystem functioning and health (15, 18, 35), and also account for the longevity composition of benthic communities, which relates to structure and biodiversity (18). The depletion and recovery parameters used to estimate RBS were sourced from meta-analyses of extensive seabed community data (7, 8) representative of the composition of benthic invertebrate communities, including primarily biomass of epi-fauna, with some infauna and count data, as well as larger- and longer-lived biota that may be more sensitive to trawling (25). We estimated recovery rates applicable to pretrawling community compositions, specifically avoiding overoptimistic assessments compromised by small, fast-growing, abundant species that may dominate the more resilient biota associated with chronically trawled areas. Furthermore, we took a precautionary approach by considering the lower uncertainty intervals for recovery rates and RBS.

Our synthesis found that the biotic status of sedimentary habitat differs greatly among regions. In most regions, some areas have low status, but large areas are little affected by trawling. Several regions, primarily in Europe, had low habitat status relative to others, highlighting where the management of trawling could be prioritized to improve seabed environmental status. A total of 20 regions have an average status >0.8, a level that has been used as an impact limit threshold for VME habitats (36). These are first-order assessments but nevertheless provide important information that can be used to broadly compare the extent of trawling effects on seabed status across multiple large-scale regions, different sedimentary habitats, and different trawl gears.

The depletion and recovery parameters are derived from meta-analyses of multiple studies spanning wide geographic areas and are generalizable given that uncertainties are also characterized. These uncertainties are substantive but are carried through to estimates of uncertainty in predicted status. Nevertheless, these parameters can be refined to reduce uncertainty and to increase local specificity. Regional assessments would benefit from analyses that were based on regionally specific gear-rigging configurations, PD, depletion rates, recovery rates, and definition and mapping of habitat types appropriate to their jurisdiction’s sustainability objectives (e.g., refs. 31, 33, and 34). Regionally determined parameters may also be able to account for additional factors, such as potential temperature, productivity, or depth effects on recovery, which were not significant in the prior meta-analysis (7). The spatial extents of our regions were also relatively large and likely to encompass substantive ecosystem heterogeneity. Ideally, ecotypes could be defined objectively at subregional scales to delimit the extent of status assessments (37). Our implementation of RBS primarily considered direct impacts on benthic communities rather than indirect impacts that may affect other ecosystem components. Nevertheless, our estimation of recovery rates from larger-scale comparative studies of benthic communities along gradients of impact magnitude on chronically trawled fishing grounds would account for indirect effects (e.g., resuspension of sediments) to the degree these impacted the sampled benthos communities. Other indirect effects are possible, potentially including impacts on trophic relationships, nutrient recycling, or demersal fishes, among others (6). However, these indirect effects are more appropriately assessed using other approaches (38); for example, food web models (6), bycatch risk assessments (39), and fishery stock assessments (38). These approaches together with RBS can provide more wholistic assessments of ecosystem state.

Our assessment of relative status for sedimentary habitats achieved widest geographic coverage with available data, but RBS is not limited to sediments or habitats. RBS can be used to assess status for particular species or taxa including VMEs (34), communities based on taxonomic groups (35), and benthos longevity classes (25). It is also possible to explicitly assess RBS for subsets of benthos contributing different ecosystem functions (15, 35). In these cases, in which continuous abundance distributions are mapped, estimates of absolute status are possible (14, 31, 34, 35) *cf* relative status as herein for habitat classes.

Unlike qualitative or categorical approaches that indicate relative risk (13, 14), RBS and other quantitative status indicators (e.g., refs. 13 and 15) enable risks of trawling on seabed status to be assessed against any defined sustainability thresholds with transparency, objectivity, and repeatability—providing guidance to support management of trawling impacts (15, 16). Nevertheless, appropriate thresholds for seabed habitats are undeveloped currently. It will require research as well as broad engagement between managers and society to define thresholds that are consistent with sustainability objectives and provide acceptable levels of precaution. RBS also enables evaluation of the effectiveness of alternative measures proposed to mitigate trawling risks [e.g., gear modifications or controls, effort limitation, and spatial management (17)]. This would be achieved by simulating their implementation and quantifying changes in predicted status. Such evaluations would facilitate management decisions involving choice of measures needed to achieve environmental objectives (14, 17, 34) and trade-offs with production (17).

We were not able to include all regions of the world where trawling occurs because of either lack of high-resolution trawl effort data or because such data were not available for confidentiality reasons (9). The formulation of policy at the national and international levels may be facilitated by wider access to trawl effort data; nevertheless, where trawling data are confidential, regional authorities can apply RBS. In regions that have only fishery-scale trawl effort data, regional SAR can be calculated from estimates of total area swept by bottom trawling divided by total regional area—and the strong relationship between regional SAR and regional RBS enables preliminary estimates of status. Importantly, this relationship also indicates that if trawl target-species exploitation is managed sustainably, the reduced regional SAR will likely lead to high seabed status. Hence, maximizing fisheries production within accepted sustainability limits and sustaining the broader environment (EAF) are complementary goals, and an objective balance between them is demonstrably achievable.

Our approaches have important implications for regional environmental and fisheries management and policy worldwide. They provide methods to address, and monitor progress toward, sustainability objectives for trawl fisheries driven by international conventions, sustainable development goals (SDGs; e.g., United Nations SDG14: “life below water”), national legislation (12), and sustainable seafood certification requirements for individual fisheries (e.g., ref. 36). RBS provides a quantitative framework that can support management decisions needed to balance fishery production with ecosystem sustainability and achieve the goals of EAF.

## Methods

### Study Objectives and Outline.

We aimed to assess the status of sedimentary habitats because these habitat types comprise the majority of seabed area, contribute to the integrity of seabed ecosystems at the broadest scales, are where most bottom trawling occurs, and lack the quantitative status assessments that managers require. We used the RBS model developed by Pitcher et al. (14) to estimate status relative to an untrawled state of biotic communities that typify seabed sedimentary habitats exposed to chronic trawling in 24 large regions worldwide where trawl footprints had been mapped by Amoroso et al. (9). Trawling impacts on seabed habitats depend on the depletion caused by different gear types, recovery rates, distributions, and exposure to trawling, thus defining the parameters and data required for quantifying the sustainability of trawling (14, 40). We estimated these parameters to implement RBS, including trawl-induced depletion rates and recovery rates, by updating a series of previous meta-analyses. Parameters were predicted for all trawl gear types (including otter trawl, beam trawl, and towed dredge) and for all combinations of percentage gravel, sand, and mud that constitute sedimentary habitats.

Trawl gear depletion rates per trawl pass were derived from trawl-impact estimates for four gear types provided by Hiddink et al. (7). We extended their existing meta-analysis to include sedimentary habitat types in addition to gear types and some additional data. The extension was based on updating the relationship between the penetration depth of gears into the sediments and the proportional rate of depletion caused by each pass of the gear, where PDs were estimated for all combinations of gear types and habitat types. Recovery rate parameters were derived by updating another existing meta-analysis by Hiddink et al. (7). We extended that analysis using a variation of their model and pooling data for both relative biomass and relative numbers as an overall measure of seabed community relative abundance, after first including some additional data. All analyses were conducted using the R Platform for Statistical Computing version 3.6.1 (41).

The wide availability of sediment mapping data enabled assessment of sedimentary habitats, which is where the majority of bottom trawling occurs. For other habitat types highly sensitive to trawling, the lack of widely available distribution data precluded RBS assessment of status herein. Instead, we estimated the proportion of each region where highly sensitive, long-lived biota types could or could not persist because of chronic trawling.

### Assessment Model.

We estimated the status of seabed habitats exposed to towed bottom-fishing gears following the RBS method of Pitcher et al. (14). RBS is based on the dynamic Schaefer (42) production model, with an additional term to describe the direct impacts of trawling on the seabed, consistent with previous seabed assessment approaches (28). The Schaefer model is commonly used in fishery assessments (e.g., ref. 43), particularly in data-poor situations in which recently it has been demonstrated to be the least biased and most frequently best performing for data-limited assessments globally (44), having excellent agreement with results from more complex models (e.g., 45 and 46). While this model is typically applied to a single species, management objectives and certification requirements also need to address seabed habitats and communities in addition to species (30, 36). Pitcher et al. (14) reasoned that while habitats do comprise many species with complex dynamics, previous studies have demonstrated that the aggregate properties of biotic communities in seabed habitats are relevant to characterizing trawling impacts (4, 5), and different sedimentary habitat types provide surrogates for their typical communities of invertebrates, which form the basis of seabed ecosystems (47). Thus, the aggregate dynamics of seabed communities in different habitats, integrated over benthos community composition and relevant time frames and spatial scales, are parsimoniously described by the Schaefer model. Furthermore, to enable application to the typically data-limited circumstances of seabed assessment, Pitcher et al. (14) took the simplifying approach that in habitats subject to chronic trawling, the long-term relative abundance of biota (*B*), as a fraction of carrying capacity (*K*), can be estimated—implicitly accounting for dynamics—by the equilibrium solution of the Schaefer model:

[1] B/K=1−F D/R where F<R/D, otherwise B/K=0,

in which *B*/*K* represents the RBS of the seabed in the range 0 to 1, *R* is the proportional recovery rate per year, which varies according to habitat, *D* is the depletion rate per trawl, which depends on gear type and habitat, and *F* is the current chronic trawling intensity as SAR (the annual total area swept by trawl gear within a given grid cell of seabed, divided by the area of that grid cell). The ratio *D*/*R* represents *sensitivity* to trawling, the time interval between trawls (years) that would cause local extinction of the biota (RBS = 0), and the ratio *R*/*D* is the corresponding *critical* F, the annual trawl SAR intensity at which a given sensitivity will have RBS = 0 (*F*_crit_). Estimating RBS requires only parameters for depletion and recovery rates and distribution maps of trawling intensity and of habitat types. These maps and the estimation of RBS within an assessed region should be determined for grid cells of size ∼1 to 3 km^2^—a scale at which the distribution of most individual trawls has been shown to be random (26, 27, 48). At larger scales among cells of this size, patterns of trawling typically are aggregated and stable over time (27, 49). Ellis et al. (28) distinguished two scales of depletion and recovery rates: *D* and *R* (as in Eq. 1) are applicable at the grid cell scale, whereas their analogs *d* and *r* are applicable at the scale of trawl gears. If trawling is distributed randomly within grid cells, then *D = d*; however, *R* < *r* and is related to *r* and *d* through the equation *R* = *rd*/[−ln(1 − *d*)] (28).

### Trawl Impact and Depletion Rates by Gear Type.

Hiddink et al. (7) and Sciberras et al. (8) conducted meta-analyses of 46 experimental studies (*n* = 152 records) of trawling impacts to estimate the proportional gear-scale depletion rate (*d*) of biota for each pass of trawls of different gear types. They used a linear mixed-effects model (lme, R package nlme) to analyze the change in biota abundance (pooled relative biomass and numbers of epifauna and infauna) with time after experimental trawling, relative to the abundance before and/or in reference areas, as log-response ratio (lnRR). Their results for the immediate log_E_ trawl impact values (*i*: the intercept of lnRR at time 0) are directly related to depletion (*d* = 1 − e*^i^*) and represent the mean estimates for each gear type across all habitat types (*SI Appendix*, Table S1). Here, we have also estimated the SEs on the natural scale and 95% CLs of the back-transformed *d* estimates for each gear type (*SI Appendix*, Table S1).

### Trawl Penetration Depth by Gear and Habitat Types.

Hiddink et al. (7) also showed that depletion rates of benthic invertebrate communities were closely related to the PD of trawl gears into the sediments. Here, we reanalyzed their data (7) for PD of each trawl gear component (i.e., doors, sweeps, and ground gear) by gear, using the same log-linear model with sediment–habitat type as a factor but including the following data updates: 1) records for Smith et al. (50) were excluded as they reported PD of their sampling gear, not trawl gear; 2) Freese et al. (51) reported PD for the otter trawl ground gear component, not whole gear, so gear-width proportion was corrected from 1 to 0.25; 3) PD data were added from Rose et al. (52) for otter trawl whole gear of 0.05 cm in mud habitat; and 4) PD data were added from Depestele et al. (53) for beam trawl whole gear of 4.1 cm in sand habitat. The final dataset comprised 71 records from 48 studies. As per Hiddink et al. (7), we aggregated the model estimates of mean PD for each gear component within gear type up to whole-gear estimates, weighted by the proportion that each component is composed of the total gear width—but whereas Hiddink et al. also aggregated across habitats, we grouped by both gear and habitat to provide separate PD estimates on the natural scale for all combinations of gear types and categorical sediment habitat types (*SI Appendix*, Table S2 and Fig. S1).

We propagated the uncertainty of the PD estimates for all gear components up to whole-gear estimates by taking 2,000 samples from the distributions of each gear-component mean, using SDs as given by the SEs of each mean reported by the fitted log(PD) model. The sampled estimates of each gear-component PD were aggregated up to whole gear-by-habitat estimates using the same procedure as for the means to provide 2,000 estimates of PD for all combinations of gear and habitat. The SDs of these estimates provide approximate SEs for PD, and the 2.5 and 97.5% quantiles provide approximate 95% CIs for PD (*SI Appendix*, Table S2 and Fig. S1).

### Trawl Depletion Versus Penetration Depth Relationship.

We estimated trawl depletion rates *d* for all combinations of gear types and sediment habitat types by refitting the same linear model of gear–mean depletion versus log of gear–mean PD relationship as figure 2 in Hiddink et al. (7) but using the updated PD estimates for both gear and habitat (*SI Appendix*, Table S2). Furthermore, because the gear–mean *d* values estimated by Hiddink et al. (7) were from varying mixtures of habitat types for each gear, for our estimates of the corresponding gear–mean PD across the three habitat types for each of the four gear types (from *SI Appendix*, Table S2), we calculated weighted mean PDs where the weights were the frequency of studies by habitat for each gear type in the experimental studies meta-analysis from which the four gear–mean *d* values were estimated (*SI Appendix*, Table S1). This was done so that the estimates of gear–mean PDs used to build the model (*SI Appendix*, Fig. S2, gray dots) would correspond to the expected PDs of the mixed habitat types represented in the meta-analysis that provided the *d* estimates.

The updated mean relationship between the depletion *d* of benthic community abundance and the PD of trawl gear was significant (*SI Appendix*, Fig. S2, gray curve, *R*^2^ = 0.98) but with uncertainty. The uncertainty of the model is indicated by the 95% CIs of the fit and by the prediction intervals (*SI Appendix*, Fig. S2, dashed gray lines and light gray shading). Additional uncertainties arise from the input data used to build the model. These include the uncertainties for the gear–mean *d*-values (*SI Appendix*, Table S1) and uncertainties for gear-by-habitat PDs (*SI Appendix*, Table S2 and Fig. S1) plus additional uncertainty arising from calculating the weighted-mean gear PD across habitats. The additional uncertainties are presented in *SI Appendix*, Fig. S2, including the 95% CIs for each gear–mean *d* from the original experimental studies meta-analysis (vertical gray lines; from *SI Appendix*, Table S1), approximate estimates of the 95% CIs for gear–mean PDs propagated using a sampling procedure (see next paragraph) (horizontal gray lines), the 95% CIs for gear-by-habitat PDs (horizontal colored lines; *SI Appendix*, Table S2 and Fig. S1), and approximate estimates of the 95% CIs for the predicted gear-by-habitat *d*-values (vertical colored lines; *SI Appendix*, Table S3), which were propagated from all sources of uncertainty using a sampling procedure (see next paragraph).

The uncertainties for each gear–mean PD were propagated by sampling the 2,000 estimates of each habitat PD for each gear type (generated as described in the previous section) in proportion to the frequency of studies by habitat for each gear type in the experimental studies meta-analysis (*SI Appendix*, Table S1). The 2.5 and 97.5% quantiles of these samples provide approximate 95% CIs for each gear–mean PD (horizontal gray lines, *SI Appendix*, Fig. S2). These uncertainties and those for gear–mean *d*-values were propagated through the model by also sampling and back transforming 2,000 estimates of each gear–mean impact *i* from the distributions of each mean using the SDs given by the SEs of each mean reported by the fitted lnRR model (*SI Appendix*, Table S1). These two sets of 2,000 sampled estimates for each gear–mean *d* and PD were used to fit 2,000 regressions, and each regression was used to predict *d* corresponding to each gear-by-habitat mean PD (*SI Appendix*, Table S2). The SDs of these predictions provide approximate SEs for each gear-by-habitat *d*, and the corresponding 2.5 and 97.5% quantiles provide approximate 95% CIs (*SI Appendix*, Table S3; vertical colored lines, *SI Appendix*, Fig. S2).

### Recovery Rates by Habitat.

In principle, experimental studies could also provide estimates of gear–scale recovery rates *r*. However, these small-scale estimates may be overly optimistic, especially for mobile fauna, due to short-distance immigration from the seabed adjacent to the experimental treatment. Grid–scale estimates of recovery are preferable. Hiddink et al. (7) observed that Eq. 1 could be used to estimate recovery rates from large-scale comparative studies of trawling effects, which sampled the expected decrease in relative abundance (*B*/*K*) of seabed communities on gradients of trawling intensity (*F*) on trawl grounds. The slope of this relationship is *D*/*R*, and if *D* is known from experimental studies, then recovery *R* can be estimated for the biotic community on trawl grounds. This approach assumes that the sampled benthos populations are approximately in a balance between trawl impacts and recovery under chronic intensities of trawling, which are known without bias (including for untrawled sites) and reflect prior trawling on ecological timescales; that “space-for-time substitution” in the studies used to derive *D* and *R* appropriately compensated for lack of prefishing historical sampling data; and that random processes and other departures are captured by the uncertainty in the relationship. These assumptions are necessary given the scarcity of recovery information, and this approach represents a significant advance for estimating the grid–scale recovery rate *R*. Furthermore, they (7) noted that trawling more rapidly depletes sensitive species and selects for species with faster life histories that are more resilient (40, 54), hence overall community *R* can be expected to increase with the intensity *F* of chronic trawling—a response Hiddink et al. (7) found was approximated by a log–linear relationship between *B/K* and *F*: log_10_(*B*/*K*) ∼ *bF,* where the slope *b* of this relationship can be used to calculate *R* using a nonlinear function of *D* and *F*.

Building on Hiddink et al. (7), we added to their data from 33 large-scale comparative studies (*n* = 677 records) of trawl impacts on benthic invertebrate communities and used an analogous meta-analysis to estimate recovery. Data for the meta-analyses were collated from published studies following a systematic review protocol (55) to eliminate bias in the selection of studies and involved a high degree of control regarding the quality of studies including specification of the trawling intensities affecting each study’s sites. We pooled data for relative biomass and relative numbers as the response ratio of overall relative abundance (*B*) since communities of benthic invertebrates comprise a combination of both biomass and numbers of a wide range of species present. Furthermore, Hiddink et al. (18) found that community biomass and numbers were the most sensitive indicators of the effects of trawling and met all nine criteria of indicator utility accepted in the literature (19). We first updated the dataset with 27 additional records for three studies (two numbers, one biomass) in gravel habitat from Collie et al. (56), and, because of the availability of improved data (57), we revised the trawling SAR intensity (*F*) for sites sampled by two studies (56, 58) (Dataset S1) and revised the sediment gravel, sand, and mud fractions for these studies and seven others with information from Asch (59) and Amoroso et al. (9), respectively. The final dataset totaled 711 records from 22 community biomass studies and 14 numbers studies and comprised 542 epifauna records and 169 infauna records, of which 539 were biomass records and 172 numbers. These data provided an extensive and representative spectrum of the composition of benthic invertebrate communities, including larger and/or longer-lived types of biota, some of which would be sessile and sensitive types.

We fitted a variation of the Hiddink et al. (7) linear mixed-effects model to estimate how community relative abundance decreased on a gradient of increasing trawling impact represented by the product *dF* of depletion and trawl intensity:

[2] $$\mathrm{lo}g_{\mathrm{10}}\left(B/K\right)∼b\;dF.$$

This variation of the model was fitted so that recovery rates of different sedimentary habitats could be estimated without confounding by trawl gear types. For each study, *d* was calculated as a weighted mean of the habitat *d* values for the appropriate gear from *SI Appendix*, Table S3, in which the weights were the respective percentages of gravel, sand, and mud fractions comprising each study’s habitat. In a second model, we added covariates for the percentage gravel, sand, and mud fractions of the habitat to estimate how the slope of the relationship changed with sediment composition (*SI Appendix*, Table S4). Overall community relative abundance decreased with increasing trawling impact *dF* (*SI Appendix*, Fig. S3*A*), with each unit increase in *dF* leading to a mean decrease in abundance of 88.1%. The rate of decrease was greater as the gravel content of the sediment increased relative to mud content.

The grid–scale recovery rate *R* was then estimated by equating Eqs. 1 and 2 for *B*/*K* and solving for *R*, giving the following:

[3] $$R=dF/\left(1-10^{\mathrm{bdF}}\right)$$

and substituting the community slope *b* estimated by fitting model Eq. 2. To account for the differing community compositions of different sedimentary habitats, the slope was varied with sediment fractions according to the coefficients in *SI Appendix*, Table S4 (*SI Appendix*, Fig. S3*B*). To account for the nonlinearity of this relationship and to estimate recovery rates of pretrawling community compositions (which include larger/longer-lived sensitive biota) rather than higher *R* values associated with chronically trawled community compositions of more resilient biota, *dF* in Eq. 3 was set close to zero (1 × 10^−9^). This approach implicitly accounts for the historical effects of prior trawling to the extent possible, and the associated uncertainty was characterized by using the SE of the slope *b* (*SI Appendix*, Table S4) to estimate 95% CIs of the mean recovery rates along sediment gradients (*SI Appendix*, Fig. S3*B*).

### Trawl Footprint and Sedimentary Habitat Mapping.

Mapping trawl footprints requires detailed information about trawling location and effort (e.g., hours of trawling); however, in most countries, such information is confidential and not publicly available. Amoroso et al. (9) approached management authorities in many regions to request access to data. Ultimately, they were provided with high-resolution information for bottom-trawl fisheries in 31 regions, including continental shelves and slopes to 1,000-m depth in Europe, North and South America, Africa, and Australasia. The information comprised a satellite Vessel Monitoring System and/or vessel logbook data encompassing a period of several years (typically three years, 2008, 2009, and 2010). For each fishing fleet, Amoroso et al. (9) also collated information about trawl gear types and sizes and towing speeds. From the product of trawling hours, gear spread width, and tow speed, they calculated SAR: the total area swept by bottom trawls each year within high-resolution grid cell locations (ca. 1 km^2^ area each) divided by the area of those grid cells. For regions where collated data coverage was >70% of total trawl effort (24 of 31 regions), Amoroso et al. (9) used grid cell SAR to estimate trawl “footprints,” the area of seabed trawled one or more times in a given region and time period. In addition, regional scale SAR can be calculated as the average annual regional total swept area divided by the total area of a region; these were also mapped by Amoroso et al. (9). The grid cell scale SAR is an area–standardized rate of trawling intensity (=*F*) that is an essential requirement for seabed status assessment. To avoid underestimating impacts, we scaled up cell *F* by 100/coverage% for each region and by gear type to approximate total trawl intensity and recalculated regional SARs and footprints (*SI Appendix*, Table S5). This scaling and recalculation assumed the collated data are representative of the spatial distribution of the total. In cases in which the unavailable data may have a different distribution, our assessment may slightly underestimate regional depletion but less so than without scaling up.

Amoroso et al. (9) also collated data for seabed sediment composition from the comprehensive global dbSEABED database of marine substrates (21) for most regions and from the MARine Sediments database (MARS, 60) for Australian regions (*SI Appendix*, Table S5). From these grain-size data (percentage of gravel, sand, and mud), they classified broad seabed habitat types to provide a consistent definition of habitat across all regions. Here, we classified and mapped regional habitats as “gravel” if gravel% > 30, else “sand” if sand% > mud%, else “mud” to match the habitats for which depletion values were estimated by meta-analyses of experimental studies (7, 8).

### Implementation of the Status Assessment.

The predicted PD (*SI Appendix*, Table S2), depletion values (*d*, *SI Appendix*, Table S3), and predicted recovery values (*R*, *SI Appendix*, Fig. S3*B*) for otter trawls, beam trawls, and towed dredges and for all possible combinations of percentage gravel, sand, and mud that constitute sedimentary habitats are presented as ternary plots in *SI Appendix*, Fig. S4. For PD and *d*, these are weighted habitat means by gear in which the weights are the percentage of gravel, sand, and mud fractions of the sediment ternary distribution—this, in effect, provides continuous estimates of PD and *d*. Recovery *R* for sediment gradients was estimated using Eq. 3 and the coefficients from *SI Appendix*, Table S4 (Model 2). The ratio *d*/*R* indicates that gravel habitats are more sensitive to trawling due to higher *d*-values and low *R*-values, sand habitats are less sensitive due to low *d*-values and intermediate *R*-values, and mud habitats have intermediate sensitivity because of high *d*-values and high *R*-values. All habitats are more sensitive to towed dredges and less sensitive to otter trawls. The ratio *R*/*d* gives the critical threshold trawling intensity (*F*_crit_) at which the estimated RBS of the average untrawled community composition would be zero (*SI Appendix*, Fig. S4).

For the 24 regions defined by Amoroso et al. (9), we used these *d* and *R* values appropriate to the trawl gear and sediment (*SI Appendix*, Fig. S4) along with grid cell SAR trawl intensity values *F* by gear type substituted into Eq. 1 to estimate RBS for each grid cell (expressed as a proportion of untrawled status between 0 and 1). The estimated grid cell RBS represents a mean estimate of the long-term relative abundance (biomass and numbers combined) of the average compositions of biota typically present in different sedimentary habitats prior to trawling as sampled by the range of studies included in the meta-analyses from which the parameters were estimated (i.e., primarily biomass of epifauna). We used the grid cell RBS values to assess the status of sedimentary habitats on the continental shelf and slope of each region (*SI Appendix*, Table S5). Trawl gear types included otter trawls, beam trawls, and towed dredges. Where more than one gear type had fished a given cell, the cumulative RBS was estimated by summing the depletion (*Fd*/*R*) due to the *d* and *F* values for each gear.

The region-wide status of sedimentary habitats, accounting for their different sensitivity and exposure to trawling by different gear types, was summarized by mapping the regional average of grid cell RBS values and by plotting the ordered distribution of grid cell RBS values (high to low) against the cumulative proportion of regional area. To capture a range of uncertainty in estimating regional RBS, the SE of the slope *b* of Eq. 2 was used to estimate lower and upper 95% CLs for recovery *R* (*SI Appendix*, Fig. S3*B*). The regional RBS distributions were calculated using the mean and lower CL for *R* to indicate the range of status for average to more sensitive compositions of biota, including larger/longer-lived types, typically present in sedimentary habitats prior to trawling. This was because of the higher level of concern for sensitive biota compared to more resilient biota that may be indicated by the upper CL for *R*.

### Relationship Between RBS and Sustainability of Trawl Fish Stocks.

For regions where stock assessment outputs were available for species targeted by bottom-trawl fisheries, we examined the relationship between average regional RBS and a measure of the sustainability of fishing on those stocks. Assuming these assessments have been implemented without significant bias, a widely accepted indicator of the exploitation status of fish stocks is the magnitude of *f* relative to the *f*_MSY_ at which fishery production is maximized over the long-term (MSY); *f*_MSY_ is considered a limit reference point, and fishing exploitation rates are considered sustainable when the ratio *f*/*f*_MSY_ < 1 (9). The mean *f*/*f*_MSY_ ratio for 2010 through 2012 was available for 87 individual trawled stocks in 12 of the 24 regions [Amoroso et al. (9), see *SI Appendix*, Table S5 for regional mean *f*/*f*_MSY_ ratios]. We plotted regional RBS against the ratio *f/f*_MSY_ and examined trends in the relationship (Fig. 4*B*).

### Status of Highly Sensitive Habitat Types.

Our primary regional RBS assessments were able to address the status of sedimentary habitats due to the wide availability of sediment data. We were not able to directly address the regional status of more sensitive habitat-forming biota types, which can form VMEs (29), because of the scarcity of large-scale distribution data for these types for all our 24 large regions. VMEs are highly sensitive to trawling because they have high depletion and slow recovery rates (29). In lieu of RBS, we estimated the proportion of each region where trawling intensity SAR was too high for long-lived habitat-forming biota to persist (if they had been present initially). Here, we define such biota as those that would have RBS = 0 at trawling SAR intensities *F* > 0.35 (i.e., with *F*_crit _= *R/d* = 0.35 and the inverse: Sensitivity = *d/R* = 1/*F*_crit_ = 2.86), which corresponds to biota types with, for example, *d*∼0.6 and *R*∼0.2—or with *d*∼0.3 and *R*∼0.1—or any other *d*/*R* ratio of about 2.86. Assuming the longevity relationship shown in figure 3 of Hiddink et al. (25), these examples would have maximum longevities of >25 and >50 y, respectively. This definition corresponds to the most trawl-sensitive of habitat-forming biota types assessed in previous case studies [e.g., >97th percentile of sensitivities for tropical taxa (31) and ∼90th percentile for temperate taxa (33)].

With this definition of highly sensitive biota, we calculated the percentage area of each region having trawl SAR *F* exceeding 0.35 where such highly sensitive biota would have RBS = 0 (*SI Appendix*, Fig. S5*A*, bars). We also calculated the percentage area of each region where sensitive biota, as defined, could persist with status > 0.8, which corresponds with where trawl SAR *F* was less than 0.07 (*SI Appendix*, Fig. S5*A*, bar colors).

We also examined the comparability of the area of regions where *F* > 0.35 with the regional trawl footprints estimated by Amoroso et al. (9) (their “uniform” approach, i.e., sum of grid cell areas *A* where *F* > 1 plus sum of *A* × *F* where *F* < 1; see symbol “|” in *SI Appendix*, Fig. S5*A*), which is indicative of a multiyear footprint. We also plotted the difference in the percentage of regional areas between these two calculations (*SI Appendix*, Fig. S5*B*). Furthermore, for each region, we also calculated the threshold *F* that would be needed to define cells as “trawled” so that the total area of those grid cells corresponds to the area of the uniform footprint (*SI Appendix*, Fig. S5*C*).

## Supplementary Material

Supplementary File

Supplementary File

## Data Availability

The trawl footprint data used in this paper were previously published by Amoroso et al. [see ref. 9, and are available as an S4 (R) object at https://sustainablefisheries-uw.org/wp-content/uploads/2018/08/PNAS-data.zip, deposited August 2018]. All other data needed to repeat the analyses in the paper are presented in the paper or the supplementary materials, or published in other cited articles.
