# Supplementary material for: Trawl impacts on the relative status of biotic communities of seabed sedimentary habitats in 24 regions worldwide
Source: Proc Natl Acad Sci U S A. 2022 Jan 4;119(2):e2109449119. doi: 10.1073/pnas.2109449119 (PMC8764683; doi:10.1073/pnas.2109449119)
Supplement: Supplementary File [file pnas.2109449119.sapp.pdf]

Supplementary Information for

Trawl impacts on the status of biotic communities of seabed sedimentary habitats in 24 regions worldwide

C. Roland Pitcher*, Jan G. Hiddink, Simon Jennings, Jeremy Collie, Ana M. Parma, Ricardo Amoroso, Tessa Mazor, Marija Sciberras, Robert A. McConnaughey, Adriaan D. Rijnsdorp, Michel J. Kaiser, Petri Suuronen, Ray Hilborn

*Corresponding author: roland.pitcher@csiro.au

This PDF file includes:

Supplementary Figures S1 to S5 2
Supplementary Tables S1 to S5 7
Supplementary Data S1. (Microsoft Excel file) 10
Supplementary References 11

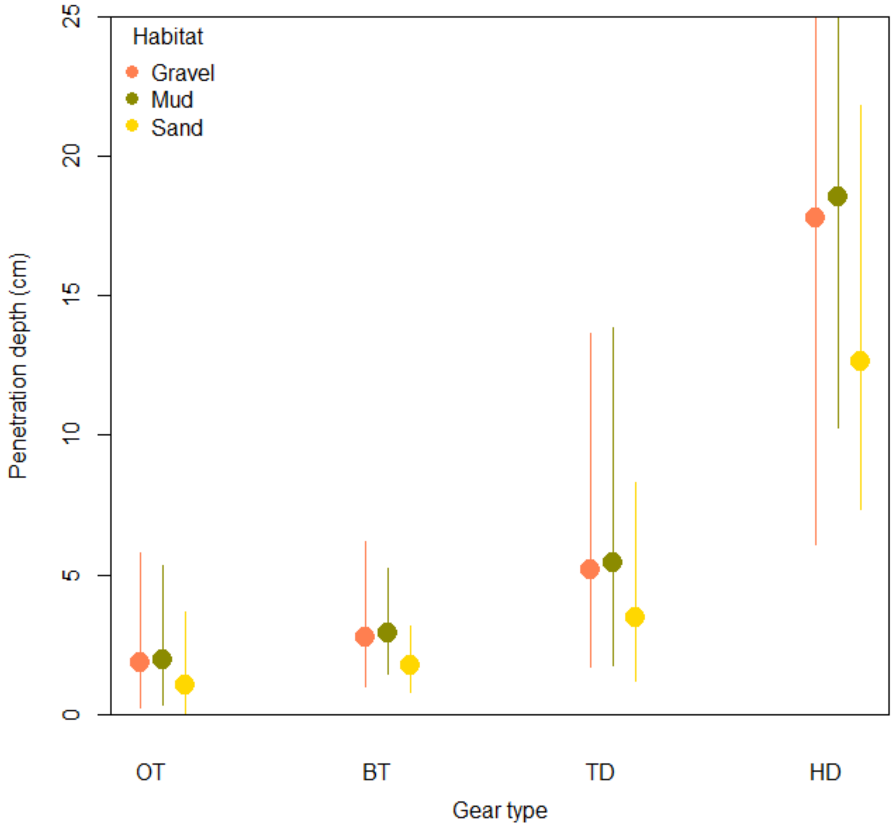

Fig. S1. Estimated trawl gear penetration depth (PD) by categorical sediment habitat types and trawl gear type, with approximate propagated 95% confidence intervals (CI), OT = otter trawl, BT = beam trawl, TD = towed dredge, HD = hydraulic dredge

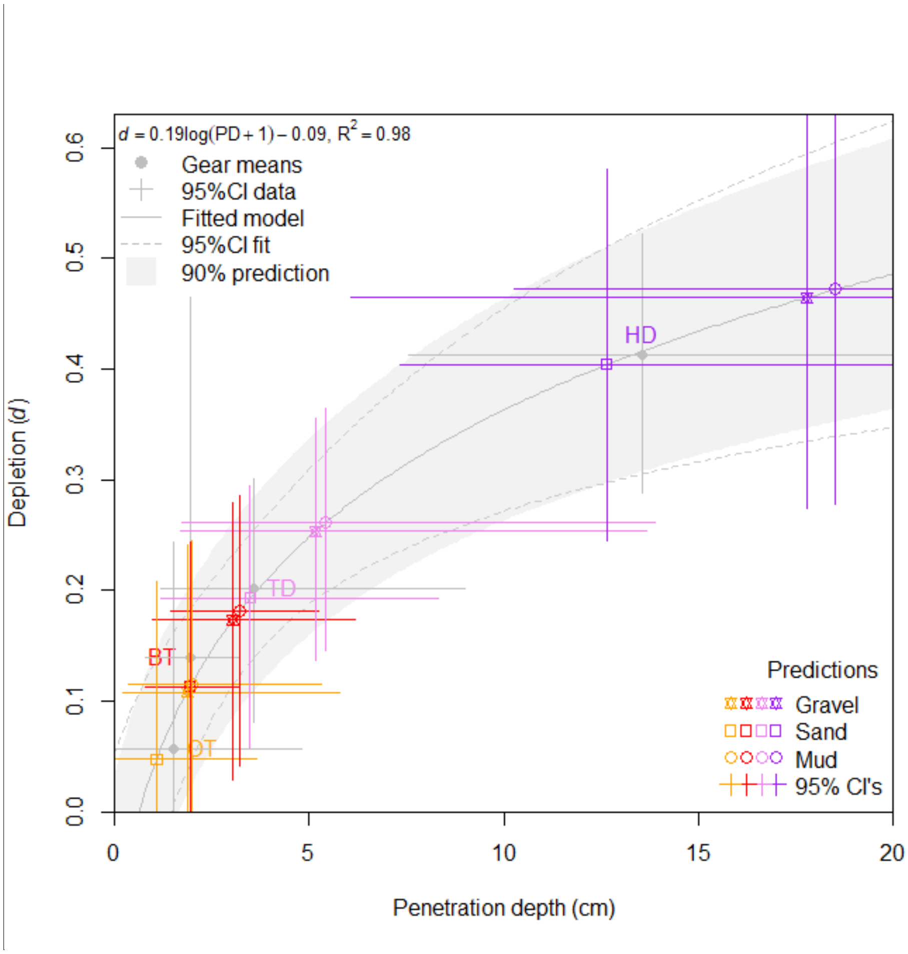

Fig. S2. Relationship between trawl gear penetration depth (PD) and proportional depletion *d* of benthic community abundance caused by a single pass of different trawl gears (means across habitat: grey points), with approximate propagated 95% confidence intervals (CI), and predictions for each sediment habitat type and trawl gear type: **OT** = otter trawl, **BT** = beam trawl, **TD** = towed dredge, **HD** = hydraulic dredge.

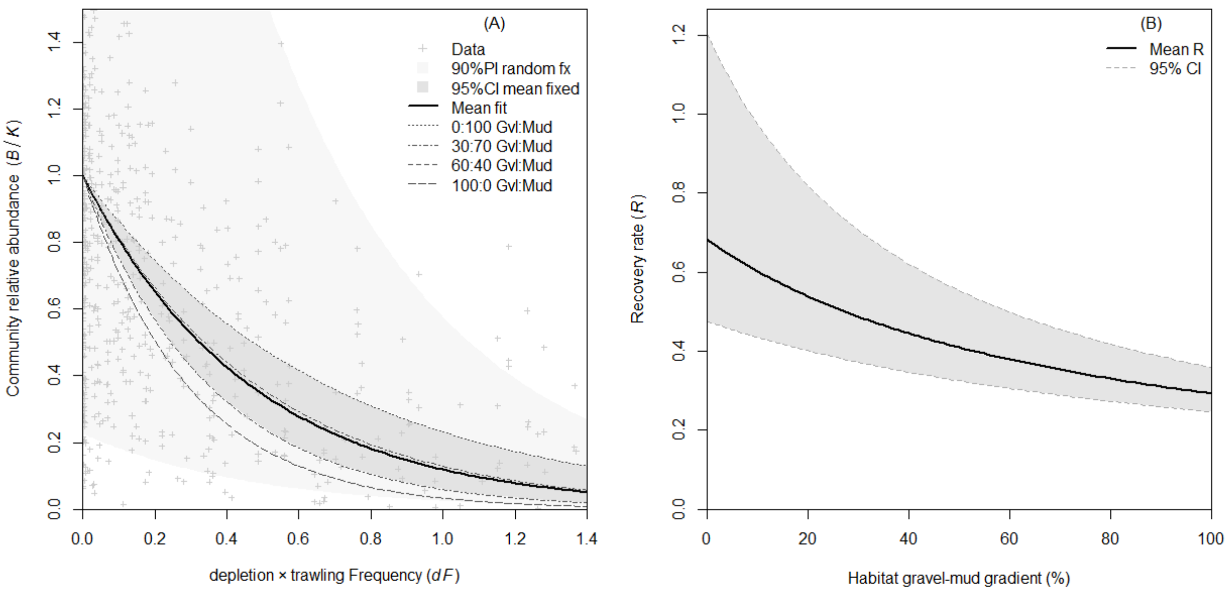

Fig. S3. (A) Relationship between overall community relative abundance and trawling impact (as the product *dF* of depletion *d* and trawl intensity *F*): the thick black line is the overall mean of the fixed effects, the darker grey shade indicates the 95% confidence interval for the mean of the fixed effects, the lighter grey shade indicates the 90% prediction probability interval of the random effects, which includes the different studies, gear types and habitat types. The dotted and dashed lines indicate how the mean relationship changes with increasing gravel:mud ratio of the sediments. (B) Relationship between predicted recovery rate (R yr^{-1}) and habitat gravel:mud ratio (ranging from 0% gravel:100% mud to 100% gravel:0% mud, with 0% sand), calculated from the slopes of the relationship in A at $dF = 1\times10^{-9}$).

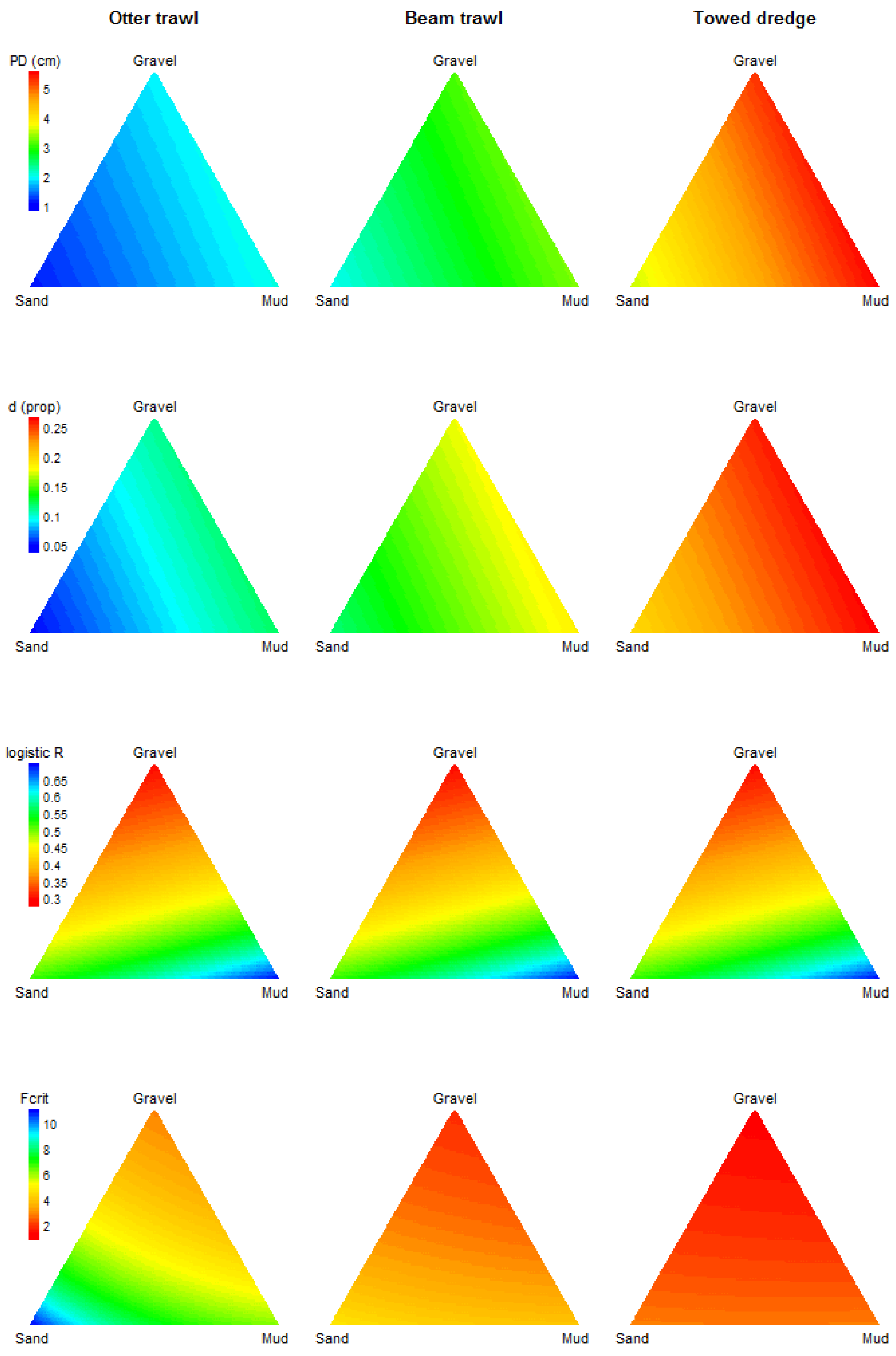

Fig. S4. Ternary plots of predicted penetration depths (PD), depletion values (*d*), recovery values (*R* yr^{-1}) and critical trawl intensity (F_{crit}, where RBS=0) for each gear type and all combinations of sediment gravel, sand and mud composition.

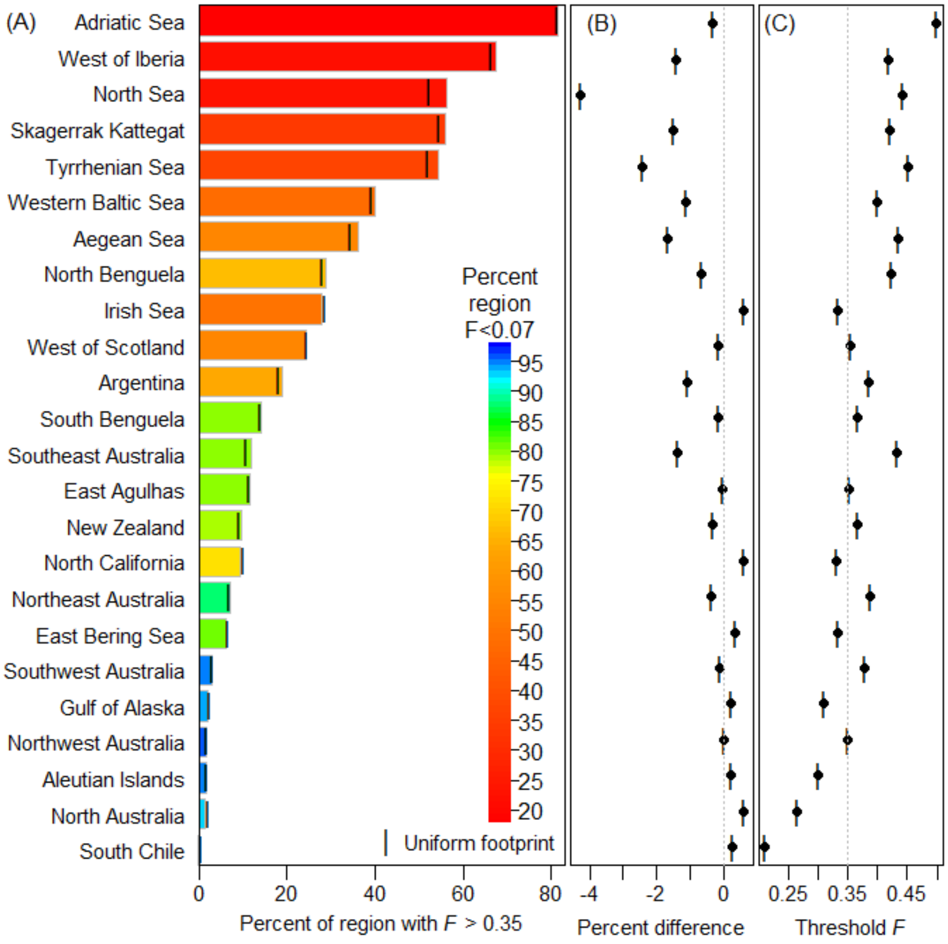

Fig. S5. Results for highly sensitive biota types. (A) Bar plots of the percentage of each region's area with trawl SAR intensity *F*>0.35, where the relative benthic status (RBS) of highly sensitive biota types (those with F_{crit}≥0.35) would be zero. The colour scale indicates the percentage of each region's area with *F*<0.07 where status of highly sensitive biota would be >0.8. The symbol '|' indicates the regional trawl footprint, as percent of each region's total area, estimated using the 'uniform' method of Amoroso *et al*. (*1*). (B) Difference between percentage area of each region with *F*>0.35 and the uniform footprint. (C) Threshold trawl SAR intensity *F* required to categorize grid cells as trawled, the area of which is equivalent to the area of the uniform trawl footprint for each region.

Table S1. Trawl impact values (*i*: as log-response-ratio, lnRR) and log standard errors (ln SE) estimated from the meta-analysis by Hiddink *et al*. (*7*), with corresponding depletion rates (*d*), back-transformed standard errors (SE *d*), and 95% confidence limits (CL), by trawl gear type: OT = otter trawl, BT = beam trawl, TD = towed dredge, HD = hydraulic dredge. Integers in columns under gravel, sand and mud indicate frequency of studies by habitats and gear that contributed to the experimental studies meta-analysis.

Gear	Gravel	Sand	Mud	Impact lnRR *i*	ln SE	Depletion *d*	SE *d*	Lower 95% CL	Upper 95% CL
OT	1	6	5	-0.058	0.111	0.057	0.105	-0.175	0.243
BT	0	4	0	-0.150	0.241	0.139	0.216	-0.388	0.466
TD	1	16	0	-0.225	0.070	0.202	0.056	0.084	0.305
HD	0	11	2	-0.532	0.104	0.412	0.061	0.278	0.522

Table S2. Estimated mean penetration depth (PD, cm), approximate propagated standard errors (SE) and 95% confidence limits (CL), by categorical sediment habitat types and trawl gear type: OT = otter trawl, BT = beam trawl, TD = towed dredge, HD = hydraulic dredge.

Gear	Habitat	estimated PD	approx. SE	lower 95% CL	upper 95% CL
OT	Gravel	1.9	1.5	0.2	5.8
OT	Mud	2.0	1.3	0.3	5.3
OT	Sand	1.1	1.0	-0.1	3.7
BT	Gravel	3.0	1.4	1.0	6.2
BT	Mud	3.2	1.0	1.4	5.2
BT	Sand	1.9	0.6	0.8	3.2
TD	Gravel	5.2	3.0	1.7	13.7
TD	Mud	5.4	3.2	1.7	13.9
TD	Sand	3.5	1.9	1.2	8.3
HD	Gravel	17.8	10.3	6.1	44.9
HD	Mud	18.5	6.0	10.3	33.2
HD	Sand	12.6	3.9	7.3	21.9

Table S3. Estimated trawl depletion rates by gear type and sediment habitat type (predicted using relationship shown in Fig. S2), with approximate standard errors (SE) and 95% confidence limits (CL) propagated from the original experimental studies meta-analysis (Table S1) and penetration depth analysis (Table S2) as well as due to prediction uncertainty from the *d* vs PD relationship (Fig. S2). Trawl gear types: OT = otter trawl, BT = beam trawl, TD = towed dredge, HD = hydraulic dredge.

**Gear**	**Habitat**	**predicted d**	**approx. SE**	**lower 95% CL**	**upper 95% CL**
OT	Gravel	0.108	0.082	-0.073	0.241
OT	Mud	0.115	0.080	-0.060	0.245
OT	Sand	0.047	0.101	-0.176	0.208
BT	Gravel	0.174	0.065	0.028	0.280
BT	Mud	0.181	0.064	0.042	0.286
BT	Sand	0.113	0.080	-0.064	0.244
TD	Gravel	0.254	0.057	0.137	0.355
TD	Mud	0.261	0.057	0.145	0.364
TD	Sand	0.193	0.062	0.057	0.295

Table S4. Coefficients of two linear mixed models for the meta-analysis of data from comparative studies of relative changes in overall community abundance (pooled response-ratio of biomass and numbers) on a gradient of trawling impact (*dF*): *d*: depletion proportion per trawl; *F*: trawling intensity as swept-area ratio. Model 1) *dF* only as independent variable; Model 2) interaction of *dF* with gravel, sand and mud fractions (%+1) of sediments. DF: degrees of freedom.

Model	Co-variate	Slope (*b*)	Std.Error	DF	t–value	p–value
1	dF	-0.9256	0.1405	686	-6.5859	0.0000
2	dF:Gravel	-0.0145	0.0029	684	-4.9348	0.0000
2	dF:Sand	-0.0083	0.0026	684	-3.1393	0.0018
2	dF:Mud	-0.0061	0.0041	684	-1.4909	0.1365

Table S5. Summary of bottom trawling footprint, annual average over typically three years 2008-2010, by region, for depths of 0-1000 m. Numbers in the first column identify regions in the figures. Codes in parentheses for European regions indicate fishery management areas. Coverage (%) of total trawling activity in each region is estimated as per Amoroso *et al*. (*9*); here trawl activity data were up-scaled 100/coverage% to approximate total trawl effort. Regional swept-area ratio (SAR) is the mean annual total area swept by trawl gears, after scaling-up, divided by the total area of the region to 1000 m depth. The uniform trawl footprint assumes that trawling is uniformly spread within grid cells and is indicative of a multi-year footprint. Sediment grain-size data (% gravel, sand and mud) were sourced from dbSEABED (*3*) or MARS (*4*) databases, from which gravel, sand and mud habitat types were classified. Relative benthic status results are the regional average of grid-cell mean RBS and the percentage (%) by area of each region with grid cell mean RBS>0.80, with RBS=0 and RBS=1.

#	Region Name	Continent	Coverage %	Area (km^2×10^3)	Regional SAR	Footprint uniform %	Sediment source	Regional mean RBS	%area of region RBS>0.8	%area of region RBS=0	%area of region RBS=1
1	Adriatic Sea (GFCM 2.1)	Europe	72	39,167	11.009	81.1	dbSEABED	0.247	20.9	68.22	17.3
2	West of Iberia (ICES 9a)	Europe	81	40,303	5.335	66.1	dbSEABED	0.596	46.4	20.85	16.1
3	Skagerrak and Kattegat (ICES 3a)	Europe	100	54,894	3.328	54.4	dbSEABED	0.633	55.1	22.60	26.7
4	Tyrrhenian Sea (GFCM 1.3)	Europe	82	137,924	2.787	51.9	dbSEABED	0.731	62.2	12.24	31.6
5	Western Baltic Sea (ICES 23-25)	Europe	72	87,070	1.282	38.9	dbSEABED	0.816	72.9	6.01	39.5
6	North Sea (ICES 6a,b,c)	Europe	86	586,108	1.215	52.1	dbSEABED	0.824	71.8	3.43	11.2
7	Aegean Sea (GFCM 3.1)	Europe	75	175,416	1.064	34.4	dbSEABED	0.834	74.4	5.06	47.6
8	Irish Sea (ICES 7a)	Europe	83	48,198	1.459	28.5	dbSEABED	0.836	80.6	9.10	17.9
9	North Benguela Current	Africa	95	203,002	1.018	28.0	dbSEABED	0.870	78.7	3.13	63.0
10	West of Scotland (ICES 6a)	Europe	81	160,640	0.506	24.2	dbSEABED	0.921	88.2	1.32	33.6
11	South Benguela Current	Africa	97	122,404	0.453	14.0	dbSEABED	0.949	91.7	0.72	70.1
12	Argentina	Americas	96	910,449	0.287	18.0	dbSEABED	0.966	96.0	0.13	54.7
13	East Agulhas Current	Africa	93	139,552	0.266	11.4	dbSEABED	0.967	95.2	0.44	61.8
14	Southeast Australian Shelf	Australasia	100	269,868	0.156	10.6	MARS	0.981	97.6	0.01	67.7
15	New Zealand	Australasia	90	1,052,723	0.118	9.2	dbSEABED	0.982	98.1	0.05	68.7
16	North California Current	Americas	100	119,327	0.107	10.0	dbSEABED	0.984	99.2	0.00	42.8
17	Northeast Australian Shelf	Australasia	100	529,357	0.129	6.7	MARS	0.985	97.9	0.17	78.6
18	East Bering Sea	Americas	97	797,969	0.073	6.4	dbSEABED	0.990	99.5	0.02	72.8
19	Aleutian Islands	Americas	97	94,721	0.026	1.8	dbSEABED	0.994	99.3	0.06	88.5
20	Gulf of Alaska	Americas	97	345,159	0.034	2.4	dbSEABED	0.994	99.4	0.02	87.7
21	Southwest Australian Shelf	Australasia	100	348,963	0.037	2.8	MARS	0.995	99.3	0.00	89.5
22	North Australian Shelf	Australasia	100	793,238	0.024	2.1	MARS	0.996	99.8	<0.01	83.8
23	Northwest Australian Shelf	Australasia	100	679,604	0.024	1.7	MARS	0.997	99.7	0.01	93.0
24	South Chile	Americas	85	188,910	0.005	0.5	dbSEABED	0.999	99.9	0.00	92.6
		**All regions**		**7,924,964**	**0.417**	**14.4**		**0.951**	**93.2**	**1.46**	**66.1**

Supplementary Data S1. (Microsoft Excel file)

Data S1. Updated trawl-gradient studies data, including additional records from Collie *et al*. (*5*) (i.e. abundance data for US study sites, and biomass and abundance data for study sites in Canadian waters of Georges Bank), and revised otter trawl and scallop dredge swept-area ratio estimates for sites sampled by Collie *et al*. (*5*) and by Smith *et al*. (*6*) on Georges Bank. The updated otter trawl and scallop dredge SAR data for US study sites were provided by Michelle Bachman, New England Fishery Management Council (*7*), and updated otter trawl and scallop dredge effort data for Canadian study sites were provided by David Keith, Department of Fisheries & Oceans. These data replace those for StudyID's = 10, 42a & 42b in Hiddink *et al*. (*2*).

Microsoft Excel
97-2003 Worksheet

Supplementary References

1. R. O. Amoroso, C. R. Pitcher, A. D. Rijnsdorp, R. A. McConnaughey, A. M. Parma, P. Suuronen, O. R. Eigaard, F. Bastardie, N. T. Hintzen, F. A. Althaus, S. J. Baird, J. Black, L. Buhl-Mortensen, A. Campbell, R. Catarino, J. S. Collie, J. H. Cowan Jr., D. Durholtz, N. Engstrom, T. P. Fairweather, H. Fock, R. Ford, P. A. Gálvez, H. Gerritsen, J. A. González, J. G. Hiddink, K. M. Hughes, S. S. Intelmann, C. Jenkins, P. Jonsson, P. Kainge, M. Kangas, J. Kathena, S. Kavadas, R. W. Leslie, S. G. Lewis, M. Lundy, D. Makin, J. Martin, T. Mazor, G. G. Mirelis, S. J. Newman, N. Papadopoulou, P. E. Posen, W. Rochester, T. Russo, A. Sala, J. M. Semmens, C. Silva, A. Tsolos, B. Vanelslander, C. B. Wakefield, B. A. Wood, R. Hilborn, M. J. Kaiser, S. Jennings*,* Bottom trawl-fishing footprints on the world's continental shelves. *Proc. Natl. Acad. Sci. U.S.A*. **115**, E10275-E10282 (2018).
2. J. G. Hiddink, S. Jennings, M. Sciberras, C. L. Szostek, K. M. Hughes, N. Ellis, A. D. Rijnsdorp, R. A. McConnaughey, T. Mazor, R. Hilborn, J. S. Collie, C. R. Pitcher, R. O. Amoroso, A. M. Parma, P. Suuronen, M. J. Kaiser, Global analysis of depletion and recovery of seabed biota following bottom trawling disturbance. *Proc. Natl. Acad. Sci. U.S.A.* **114**, 8301-8306 (2017).
3. C. J. Jenkins, Building Offshore Soils Databases. *Sea Techn.* **38**, 25-28 (1997).
4. V. Passlow, J. Rogis, A. Hancock, M. Hemer, K. Glenn, A. Habib, "Final Report, National Marine Sediments Database and Seafloor Characteristics Project" (Geoscience Australia, Record 2005/08, 2005).
5. J. S. Collie, J. M. Hermsen, P. C. Valentine, F. P. Almeida, "Effects of fishing on gravel habitats: assessment and recovery of benthic megafauna on Georges Bank" in *Benthic habitats and the effects of fishing* P. Barnes, J. Thomas, Eds. (American Fisheries Society, Bethesda, MD, 2005), Vol 41, pp. 325-343.
6. B. E. Smith, J. S. Collie, N. L. Lengyel, Effects of chronic bottom fishing on the benthic epifauna and diets of demersal fishes on northern Georges Bank. *Mar. Ecol. Prog. Ser*. **472**, 199-217 (2013).
7. M. Bachman, P. Auster, J. Coakley, G. DePiper, K. Ford, J. Livermore, D. Packer, C. Quartararo, D. Stevenson, P. Valentine, A. Verkade, F. Restrepo, T. S. Smeltz, "Fishing Effects Model Northeast Region" (Final Report, New England Fishery Management Council, 2019); https://www.nefmc.org/library/fishing-effects-model
